# What are the principles that govern life?

**DOI:** 10.1080/19420889.2020.1803591

**Published:** 2020-08-10

**Authors:** Jaime Gómez-Márquez

**Affiliations:** Department of Biochemistry and Molecular Biology, Faculty of Biology - CIBUS, University of Santiago de Compostela, Galicia, Spain

**Keywords:** Life rules, vital determinism, evolution, cooperativity, reformulated central dogma, entropy and life

## Abstract

We know that living matter must behave in accordance with the universal laws of physics and chemistry. However, these laws are insufficient to explain the specific characteristics of the vital phenomenon and, therefore, we need new principles, intrinsic to biology, which are the basis for developing a theoretical framework for understanding life. Here I propose what I call the seven commandments of life (the Vital Order, the Principle of Inexorability, the reformulated Central Dogma, the Tyranny of Time, the Evolutionary Imperative, the Conservative Rule, the Cooperating Thrust) as a set of principles that help us explain the vital phenomenon from an evolutionary perspective. In a metaphorical way, we can consider life like an endless race in which living beings are the runners, who are changing as the race goes on (the evolutionary process), and the commandments the rules.

## Introduction

In the last two centuries, there has been enormous scientific progress in the understanding of biological processes. Currently, Biology is entering a new phase focused on the analysis of immense amounts of information that allow us to address the study of complex systems such as the genomes or the brain, or even reveal the mystery of the origin of life. However, despite such huge amount of information and the new methodological and analytical tools, we still need to elaborate a conceptual framework to answer the fundamental questions about the nature of life.

Physics and chemistry have laws and theories to explain the universe but biology does not. The reductionist perspective that the laws of physics and chemistry are sufficient to explain everything that happens in living organisms does not, however, provide a satisfactory explanation of vital phenomenon. Through the last centuries, biologists, physicists and philosophers have tried to formulate the universal principles that govern life. In 1944, E. Schrödinger in his book *What is life?* wrote: “Living matter, while not eluding the laws of physics and chemistry as established up to date, is likely to involve other laws of physics hitherto unknown, which however, once they have been revealed, will form just as integral a part of science as the former” [1]. The biochemist N. Lane in his book *The Vital Question* states that there is a black hole at the heart of biology because we do not know why life is the way it is and we do not know that because we still do no have the conceptual tools to understand life as a whole [2]. Physicist P. Davis in *The 5th Miracle* also reflects on the meaning of life and concludes “True progress with the mystery of biogenesis will be made, I believe, not through exotic chemistry, but from something conceptually new” [3]. Science philosopher C. Cleland in her book *The Search for a Universal Theory of Life* offers an accurate analysis of the challenges of formulating a universal theory of life [4].

## The commandments

The philosopher E. Kant argues that all natural phenomena are law-governed: “Everything in nature, both in the lifeless and in the living world, takes place according to rules, although we are not always acquainted with these rules” [5]. That life must conform to the laws of physics is an absolutely true statement [6]. But the question is whether those laws are sufficient to explain the vital phenomenon. It is at this point that discrepancies can exist between scientists or philosophers. I believe that in addition to the universal laws of science, we need a specific conceptual framework to explain and understand the phenomenon of life.

The best example of a theory in Biology is undoubtedly the theory of evolution of Ch. Darwin [7,8] and A.R. Wallace. Despite the success of his theory, Darwin never formalized it in mathematical terms; he was aware of this formal gap that could weaken his brilliant theory of evolution [9]. What was revolutionary in this case was the concept of evolution applied to living beings even though it lacked mathematical support. Many years after this brilliant idea, another great scientist, F. Crick, enunciated the central dogma of molecular biology [10] and thus laid the foundations of modern biology.

**Figure 1.**
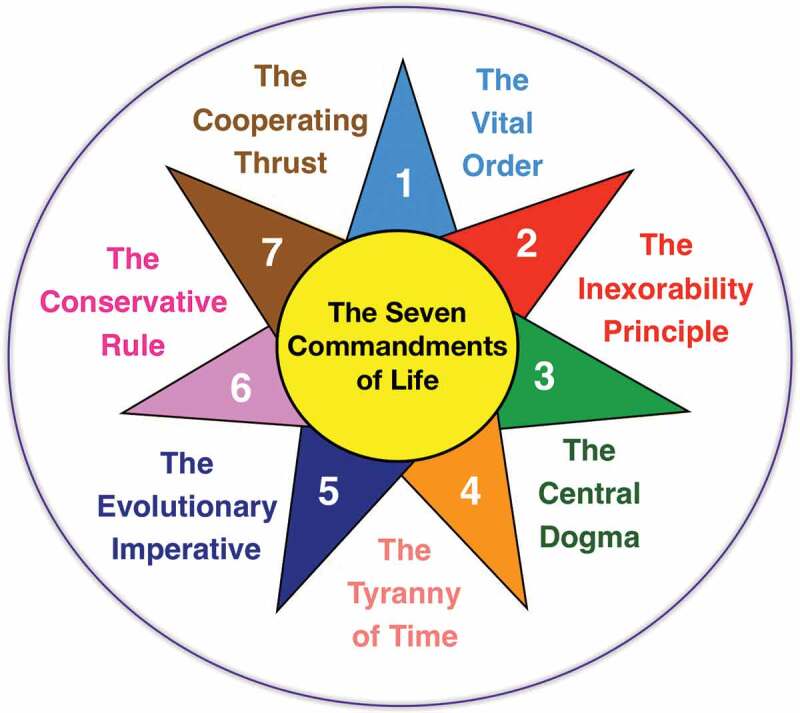
The seven commandments of life. The commandments are metaphorically represented as a sun with seven rays that help to better understand the vital phenomenon.

Here I propose what I call the seven commandments of life (outlined in Figure 1) as a set of principles that may help us to understand the vital phenomenon and the evolution of organisms in nature. To elaborate the set of commandments I tried to unite and organize some of the things we know about the phenomenon of life and combine them with my own ideas. I do not dare to call them laws because they were not obtained from the standpoint of mathematics, are the fruit of my reflections and experience in teaching and research, as well as the contributions of other scientists and philosophers.

## The vital order

Reflecting on life, the first thing that came to my mind was something obvious: living organisms are highly ordered structures and only disorder appears with illness, aging and death. Without order, the chemistry of life can exist, but life cannot. This is how the first commandment, the Vital Order, came about.

This commandment states: only life can create life. Already in the seventeenth century, F. Redi stated something like this with the sentence *Omne vivum ex vivo* [11]. The vital order means that only a living thing can create another living thing and this is so because organisms are highly ordered structures alive, and if this vital order is lost, life cannot be created. A simple demonstration of this can be seen in the in vitro cell culture: if we culture cells under appropriate conditions, cells will divide normally; however, if we break them down (we disorder them), life disappears and no new cells will be produced although all cellular components were present. Therefore, from disorder life cannot arise except at the origin, where life probably arose from a disordered prebiotic soup. A. Szent-György wrote about the mystery of life: “My own scientific career was a descent from higher to lower dimension, led by a desire to understand life. I went from animals to cells, from cells to bacteria, from bacteria to molecules. … On my way life ran out between my fingers” [12]. This beautiful metaphor illustrates very well the first commandment: when order was lost in the transit of bacteria to molecules, life disappeared and we could not see life any longer, we could only see the molecular components of a disorganized living cell.

The first commandment is closely related to the second law of thermodynamics, but it has its own path as a biological principle. The relationship between life and entropy was recognized by L. Boltzmann in 1886: “The general struggle for existence of living beings is therefore not a fight … for energy … Rather, it is a struggle for entropy … ” [13]. We know that living beings do not violate the second law because they keep their entropy low by increasing the disorder in the environment causing a net increase in entropy [14]. To keep this low entropy and to perform biological work, living systems need an external energy source. Certainly, the strategy to obtain energy to keep the entropy low was one of the main conditioners of evolution.

Isolated systems spontaneously evolve toward thermodynamic equilibrium, the state with maximum entropy. However, living organisms are open systems and life is a far-from-equilibrium thermodynamic process [15]; if a living being reaches equilibrium with its surroundings, then the quality of life disappears. Living organisms face changes every moment of their lives and require a constant energy input to maintain their highly ordered state. In this vital process the only thing that remains unaltered is the vital order and the non-equilibrium state; if the vital order is lost then the whole biosystem goes to an irreversibly state that we call death.

Reproduction is the victory of life over entropy because it generates a new vital order. Once an organism is born, it begins a race against the arrow of time and finally succumbs to the second law of thermodynamics at death. In the struggle of which Boltzmann spoke there are two winners: life, because reproduction generates a new vital order, and entropy because the activity of living beings and the decomposition of living matter after death produces an increase in the entropy of the universe.

## The inexorability principle

Observing the vital phenomenon from the molecular level to the ecosystems, I came to the conclusion that there is a kind of determinism in the way things happen. For example, if we analyze the genetic code and the central metabolic pathways we see that they did not change for millions of years and if we look at nature on the macroscopic level we can also see a great deal of conservation and convergence in complex processes as embryonic development or anatomical and physiological adaptations. From this simple reasoning arises the second commandment, the principle of inexorability, which can be summarized as follows: “life is like that because it should be like that“. This kind of vital determinism, which has nothing to do with the philosophical doctrine known as Vitalism [16], means that every structure and every biological process, from the molecular level to the ecosystems, is as we know it because it must be like that with small variations. However, it should be noted that the principle of inexorability does not mean that there is an evolutionary determinism in life. There is no a predetermined plan of what nature has to be and what we can see is the result of millions of years of constant trial-error experiments conducted by the laws of nature. For example some organisms developed eyes because their presence is a requisite for the vision (natural selection selected the best-adapted organisms by choosing the adequate genomes), not because there was a predestination to have eyes. In the history of life, this commandment together with the evolutionary imperative (the fifth commandment) was very important in the configuration of nature. The principle of inexorability is so important in the history of life that I will describe some examples to demonstrate its validity.

Proteins play many different roles in every cellular process and they need to fold properly to carry out their function. Considering a small protein (100 amino acids), C. Levinthal calculated that the total number of hypothetical structures would be 3^100^ and if it takes 10^−13^ s to convert one structure to another, the total search time to find the right structure would be 1.6 × 10^27^ years. However, a protein needs less than 1 s to fold properly. This enormous difference between both times is known as the paradox of Levinthal and shows that proteins must follow a defined folding pathway [17,18]. Therefore, there is a chemical-physical determinism in protein folding and any mistake in this process, generating wrong folding patterns, would be probably lethal for the cell.

We can find in nature complex self-assembled macromolecular structures such as virus, ribosomes, signal receptors or multi-enzymatic complexes. As we observed with proteins, we can see a molecular determinism governing the correct and fast assembly of these macromolecular complexes. To explain this I choose two well-known examples: bacteriophages and ribosomes. The assembly of λ bacteriophage infectious particles occurs inside bacteria and involves several specific interactions protein-protein and protein-DNA [19]. Well, packaging and maturation of λ DNA to form virions can also take place in cell-free extracts where λ heads and tails assemble independently and spontaneously join *in vitro* [20]. The ribosome assembly is an important research topic in molecular systems biology since ribosomes are complex molecular machines composed of 3–4 different rRNAs and 55–79 proteins [21,22]. In spite of this complexity, *in vitro* assembly or reconstitution of *Escherichia coli* ribosomes from purified native components can also be achieved in the test tube [23]. Both experimental results strongly support that there is a molecular imperative that drives the construction of big macromolecular structures inside the cells.

The paradigm that a metabolic pathway could only occur in the presence of enzymes changed when it was shown that glycolysis and pentose phosphate pathway-like reactions could take place in a plausible Archean ocean in the absence of enzymes [24]. Subsequently, it was reported a non-enzymatic promotion of multiple reactions in which pyruvate and glyoxylate build up most of the intermediates of the Krebs and glyoxylate cycles [25]. These results demonstrate the existence of a metabolic determinism and support the prebiotic genesis of metabolism.

At the multicellular level, we can also see many evidences of this commandment and a good example of this is convergent evolution. There are many examples of convergent evolution in nature such as the evolution of complex eyes in vertebrates, cephalopods and arthropods, the echolocation system in whales and bats, the evolution of woody stem in seed plants, horsetails and trees, the silk producing ability of spiders, silk worms, silk moths and weaver ants, wings, etc [26]. Interestingly, it was found that increases in the hemoglobin-oxygen binding affinity occurred in different alpine species (convergent evolution), but the molecular changes underlying in the hemoglobin molecule were variable and unpredictable revealing that convergent adaptive traits can also arise from different genetic changes [27]. As Conway Morris said “life will inevitably evolve towards an *optimum* body plan” [28].

Is the Inexorability Principle related to the “Intelligent Design” [29]. The answer is no because nature is neither the consequence of an intelligent designer nor a prior design of what nature is supposed to be; what we observe is the result of millions of years of evolution driven by the laws of nature. On the other hand, is the Inexorability Principle related to Causal Determinism? If we define determinism as “the world is governed by (or is under the sway of) determinism if and only if, given a specified way things are at a time t, the way things go thereafter is fixed as a matter of natural law” [30], the answer would be no because in the evolutionary process both determinism and contingency (chance) play a role in the evolutionary process. An example that illustrates very well this point is related to the evolution of echinoids [31]. Thus, all echinoids (sea urchins, sand dollars, etc.) are descended from one or two species that survived the great End-Permian mass extinction. As it happened, this one group had two columns of plates in the test. Consequently all the descendants also have these two rows of interambulacral plates, while in Permian species the number of rows of such plates varied from one to eight. As D. Erwin says “one can argue that the group with two plates was somehow better adapted, or that they simply survived by chance. In truth, either possibility is equally likely” [31]. From my point of view, the condition of having plates represents the vital determinism (the inexorability principle) but whether they have 1, 2 or n plates are contingency.

S. J. Gould asked what would happen if we “replay the tape of life” [32]. My answer to this question is that life would be very similar to what we know (fossils and extant creatures) providing the environmental conditions were about the same: for vision, living beings would develop eyes, to fly, wings, or to harness the energy of the sun, photosynthesis. In other words, organisms evolving under similar ecological conditions often evolve similar traits. There are many studies that reinforce this idea of repeatability of evolution. For instance, there is the case of cichlid fishes in lake Malawi and lake Tanganika who developed strikingly similar body shapes [33], or the three distantly related lineages of snakes that have convergently evolved resistance to tetrodoxin found in their prey via the same amino acid substitution in the Na^+^/K^+^ ATPases [34].

An important corollary of this commandment is that if there is life elsewhere in the universe it should be very similar to what we know on Earth and the differences between the Earth living forms and the “space creatures” could be attributed to a different evolutionary stage or to specific environmental conditions. In addition to the principle of inexorability, I am convinced that it should be so because the laws of physics are the same throughout the universe and the matter of stars contains the same atoms found on Earth although in different proportions. Thus, providing that the environmental conditions are similar, on a distant planet if there is glucose in a watery medium it will probably end up turning into pyruvate, proteins will fold up like here, radiant energy would be transformed into chemical energy by a mechanism similar or identical to photosynthesis, or if an organism had to fly it would have wings. And so on. This hypothetical premise could be very important when developing projects that seek life elsewhere in the universe.

## The reformulated central dogma

**Figure 2.**
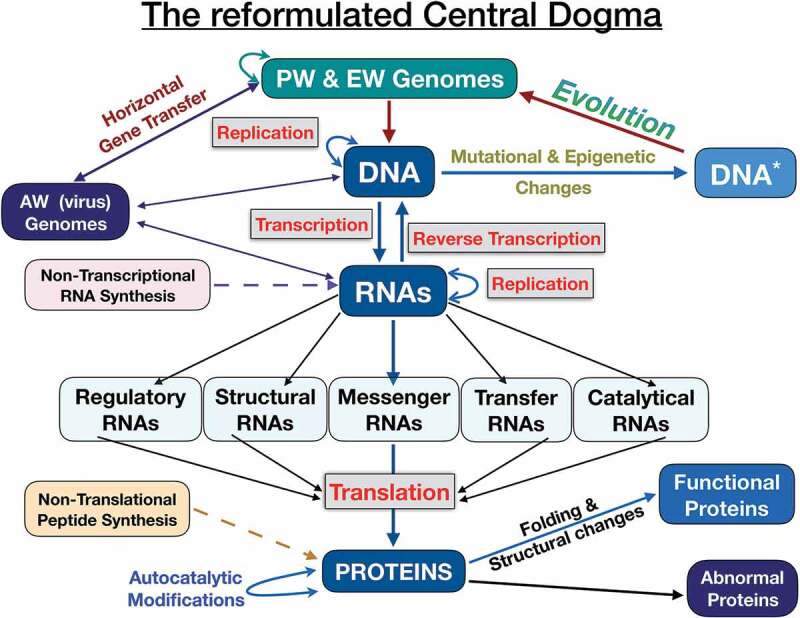
The reformulated Central Dogma. This scheme is a variation of the original version by F. Crick [10]. The genetic material of prokaryotes and eukaryotes is DNA whereas in viruses is either DNA or RNA. DNA can mutate and undergo epigenetic changes and this altered DNA is the target for evolution. Horizontal gene transfer also plays an evolutionary role in the flow of genetic information between species. RNAs play a central role in the flow of genetic information because link DNA (information) with proteins (cellular actions). Noteworthy, all different kinds of RNAs are involved in translation. RNA and proteins can be synthesized by transcription and translation, respectively, and also by a non-transcriptional and non-translational mechanisms. Proteins undergo folding and post-translational modifications to become a functional protein. Some proteins have the capability of autocatalytic modifications.

We know that the molecular mechanisms involved in genome expression and replication are universal. This makes it possible to replicate, transcribe or translate, for example, a human gene (from the eukaryotic world) into a bacterium (from the prokaryotic world), or a viral gene (from the acellular world) into a eukaryotic cell. The central dogma of molecular biology was first enunciated by F. Crick [10] and tells us about the flow of genetic information from DNA to RNA to proteins. This orderly transmission of genetic information has not changed in millions of years, which is proof of biological soundness and evolutionary importance. It was these considerations that led me to propose a reformulated version of the Central Dogma as a third commandment, but on the basis of our current knowledge of molecular biology. Figure 2 shows the scheme of the reformulated Central Dogma.

Since the central dogma was proclaimed for the first time, they were discovered new kinds of RNAs, epigenetic changes in DNA, new enzymes, etc. Crick himself anticipated it might be incomplete: “The central dogma … is likely to prove a considerable over-simplification” [10]. All these new discoveries have enriched the central dogma and have also served to correct some inaccuracies in the premises of the primitive dogma [35–37]. This new scheme shows that genetic information flows from DNA to DNA (replication, epigenetic changes, mutations, horizontal gene transfer), from DNA to several kinds of RNAs (transcription) and from RNA to DNA (reverse transcription), from RNA to RNA, from RNA to proteins (translation). The original Central Dogma is invalid as an ‘absolute’ principle because transfer of information from proteins (prion-mediated inheritance) to the genome does exist [38]. As E. Koonin wrote this is not to deny that the classical Central Dogma does capture the principal route of information transfer in biology [39].

In the scheme shown in Figure 2, RNA occupies a central position linking DNA and proteins and showing its multifunctional roles (regulatory, catalytic, structural, informative and transport) [40]. It is worth noting the catalytic role of RNA (self-splicing, tRNA processing, peptide bond formation) because it shows that enzymatic activities are not exclusive of proteins. The multiple functions of RNA molecules provide strong evidence for its predominant role in the cell biology as well as in the origin of life in a hypothetical prebiotic “RNA world” [41].

Finally, I have also added two new enzymes that could have played a fundamental role in the prebiotic world and that have been preserved to this day: polynucleotide phosphorylase (PNPase) and non-ribosomal peptide synthetases (NRPS). PNPase is an evolutionary conserved bifunctional enzyme with a phosphorolytic exonuclease activity and the capacity of synthesizing RNA using any ribonucleoside diphosphate [42]. PNPase synthesizes RNA without any of the components involved in transcription and for this reason, I call this as non-transcriptional RNA synthesis. PNPase also plays a pivotal post-transcriptional regulator function in both bacteria and humans. NRPS are complex molecular machines that synthesize small peptides with powerful biological activities [43]. NRPS are widely distributed in bacteria and found sporadically in archaea and eukarya. NRPS synthesize non-ribosomal peptides that can contain proteogenic and non-proteogenic aminoacids and since translational machinery is excluded in this synthesis I call this as non-translational peptide synthesis.

We know that evolution of species occurs because genomes can change by chance and necessity using the words of Monod [44]. However, the backbone of Central Dogma and the genetic code (with few exceptions) once established has remained unchanged over millions of years of evolution. The Central Dogma is not a fixed, immutable photograph, but the embodiment of an evolutionary reality that represents the changes in the biology of genomes but without altering their essence and beauty. 

## The tyranny of time

If we look around us we see that all living beings are perishable, that the passage of time leads to our disappearance. We are born, we grow, we age and we die and all this happens in a time sequence, sometimes established with great precision. There is little we can do against the passage of time and that is why I speak of the Tyranny of Time and include it as the fourth commandment.

This commandment says that there is a submission of life to time. Time determines life but life cannot change time because time is usually not made up of, or dependent on, anything else. Time is so important that almost every vital process has its own time and all living organisms show a behavior that indicates awareness of time [45]. For example, cell cycle always occurs in a precise space-time sequence and to achieve this precision, thousands of molecules must unconsciously cooperate to pass each phase of the cycle in time to division (this is another example of the inexorability principle).

As physics shows us there is a close relationship between entropy and time and the flow of time is inherent in the second law of thermodynamics. A. Eddington coined the phrase “arrow of time” to illustrate the directionality in time which means that as time progress entropy increases [46]. Life is order and time plays against this order because of the arrow of time. Therefore, life needs free energy to struggle against entropy (it cannot fight against time) and to maintain the vital order.

Time leads to disorder but it is necessary to reach order. This is what I call the “Paradox of Time” and it can be formulated as follows: “what time makes possible, time makes impossible”. It is easily conceivable that in the origin of life it was necessary a long time to generate the prebiotic soup (time make it possible) but the formation of the first cell had to be instantaneous otherwise disorder would triumph (time would make it impossible). Each new living being has to fight against the arrow of time, without knowing that because the tyranny of time it will always loose this battle. The only way of overcoming the tyranny of time is to reproduce and set the life timer to cero. 

## The evolutionary imperative 

Evolution is past, present and future. Evolution is among the most substantiated concepts in science and represents the unifying theory of Biology [47]. This idea is perfectly summed up in the phrase of T. Dobzhansky: “Nothing in biology makes sense except in the light of evolution” [48]. The fifth commandment speaks of the Evolutionary Imperative and it is an imperative because if life did not evolve it would eventually disappear. Evolution is inherent in life and has shaped biodiversity over billions of years since the first cell was formed. Evolution is necessary to overcome the environmental and biological changes that may occur in nature. Evolution is life and life is evolution.

There are four classical evolutionary forces: natural selection, genetic drift, mutation and migration [47]. Natural selection is defined as the process of adaptation of an organism to its environment by means of selectively reproducing changes in its genotype; it is like a pressure that causes populations of organisms to change over time. Genetic drift and migration are random processes in which chance plays a role in deciding which gene variants survive. Mutations are changes in the genetic material and when they are advantageous they are selected, fixed and passed to the next generations. The need to evolve is embodied in the genomes whose changes are the source of variability for the evolutionary forces to act. Nevertheless, I think that these evolutionary forces are not enough to understand the evolutionary process because, at most, they can explain how complex systems evolve but they do not provide an explanation of why organisms are the way they are, and not some other way. In this sense, I want to put forward a recomposition of the evolutionary forces by adding to the classical ones the vital determinism (understood as the consequence of the inexorability principle) as well as the interactions between the biotic (acellular, prokaryotic and eukaryotic) and abiotic (environmental) worlds.

We know that natural selection is a fundamental mechanism in the evolutionary process because it causes species to change and diverge over time. Organisms that are better adapted to their environment are more likely to survive and pass on their genes to their offspring. In this natural process, how does vital determinism fit in? Even though there is no determinism in life because evolution has no sense of the future and it has no pre-established goal, every living being will inevitably tend to adopt the characteristics necessary to succeed in nature and here it is the vital determinism. An example of this combined evolutionary force is the acquisition of wings to fly by evolutionary distant animals (pterosaurs, insects, birds, and bats) [49]. The wings are not modified versions of a structure present in a common ancestor but rather they have developed independently. There was no evolutionary pre-determinism that imposed the existence of winged animals; what happened is that some animals developed wings and were selected because they are the best aerodynamic solution to fly (vital determinism), otherwise unrelated species could have developed different alternatives for the same purpose.

Another example of combined vital determinism and natural selection is how different plants in distant places found the same solution for the same problem. New world cacti and African euphorbias are alike in overall appearance (both are succulent, spiny, adapted to arid conditions); although they belong to separate families, their morphologies have evolved similarly and independently in response to similar environmental challenges [50]. The example of cactus and euphorbias illustrates very well the joint action of the principle of inexorability (vital determinism) and natural selection. It is the second commandment that “compelled” these plants to have evolved similarly and independently in response to similar environmental challenges, and it is natural selection that took care of eliminating the worst adapted individuals. The combination of vital determinism and natural selection could help to better understand convergent evolution as well as the process of speciation.

Genome modifications (duplications, transposition, point mutations, insertions, deletions, chromosomal translocations and inversions, exon shuffling, genome reduction, epigenetic changes, horizontal gene transfer and recombination) are the source of variability necessary for the evolution of species. Moreover, random events (mutations, genetic drift, migration) generate genetic variability by chance. J. Monod in his book *Chance and Necessity* [44] supports that life is only the result of natural processes by “pure chance”. This is only partially true because chance or contingency play an important role in evolution but vital determinism and nonrandom processes, such as recombination or epigenetic changes, also contribute to genomic changes. A proof of this was the recent report showing that there is coordination between stochastic and deterministic specification in the neurodevelopment of *Drosophila* visual system [51].

All species that make up the ecosystems are, directly or indirectly, interconnected to each other and with their environment. The interactions amongst the three natural worlds (acellular, prokaryotic and eukaryotic) with their environment were basic in evolution. We can see many types of interaction between the worlds, such as cooperative interactions like symbiosis, infections and diseases, horizontal gene transfer, etc [52]. What happens to one species ends up affecting others. The ensemble of worlds maintains the dynamic equilibrium in the ecosystem that simultaneously has the plasticity to evolve. 

## The conservative rule 

The sixth commandment means that life always preserves what is good for life or, in other words, once the evolutionary process finds and selects a structure or a process that works well at any level of complexity (from macromolecules to multicellular organisms) it will not change it or if it does it would consist only in a fine-tuning. There are multiple examples of this conservative rule in nature at very different levels: the universality of genetic code, proteins whose amino acid sequence or tridimensional conformation did not change, the basic metabolic pathways, the presence of wings in flying animals, the eye evolution, the anatomy of a vascular plant, etc. This commandment is closely related to the second and fifth commandments and altogether drove evolution toward the best stable solution for every biological challenge. The joint action of these commandments created the beauty and perfection that we can observe in nature.

## The cooperating thrust 

**Figure 3.**
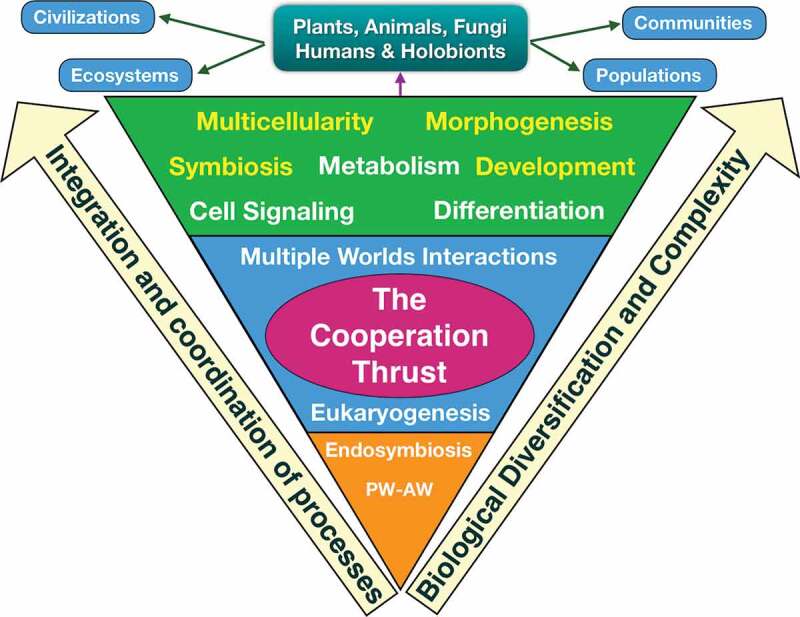
The cooperating thrust. Since life on earth originated, the cooperative impulse has played an essential role in two fundamental evolutionary processes: eukaryogenesis by endosymbiosis and the generation of multicellular organisms, which involved biological diversification (generation of plants, animals, fungi), an increase in the complexity of organisms (tissues and organs) and the emergence of mechanisms for the integration and coordination of biological processes involved in development, morphogenesis, cell signaling, etc. Symbiosis gave rise to new forms of survival and in some cases such close symbiotic relationships were established that they led to the emergence of holobionts as an evolutionary unit. The multicellular eukaryotic organisms were organized in populations, communities, ecosystems and the *Homo sapiens*, due to the development of the brain, gave origin to the civilizations.

The seventh commandment, the Cooperating Thrust, is about the need for cooperation as a survival-reproductive strategy. There is cooperation everywhere in nature from the molecular level to symbiotic and social interactions, and in the past, it was involved in two of the most important transformations on the history of life: eukaryogenesis and multicellularity (Figure 3). We can see cooperation in the metabolism or in the expression of DNA to manufacture proteins, endosymbiosis and symbiosis, cooperation amongst different types of cells like in the immune system, or cooperative organizations to build societies (humans and insects do that). In the evolutionary process the rise of cooperative organization was fundamental to conquer the world [53,54]. Biofilms are microbial communities that form on heterogeneous surfaces [55]; they represent a very interesting example of cooperation and are a *bona fide* proof that cooperativeness was present early in evolution. It is worth noting that some biofilms consist of microorganisms of the same species and other are very diverse taxonomically involving organisms form the three worlds (viruses, archaea, bacteria, fungi and unicellular eukaryotes) [56]. Biofilms are a strategy of survival and resemble a primitive form of multicellularity. On the other hand, we can see cultural and technological evolution giving rise to many civilizations throughout the history of human race.

Symbiosis defined as any of several living arrangements between members of two different species is presently recognized as one of the main forces shaping life in our planet [57]. At present we can observe many ways of symbiotic cooperation (positive or beneficial and negative or harmful) between species belonging to the same or different worlds (coral reefs, ants and fungi, lichens, luminescence organs, gastroinstestinal flora, sea anemones and hermit crabs, African oxpeckers, nitrogen fixation, etc.). It is a reflection of different cooperation ways, an example of permanent interactions between species from the three worlds. I want to highlight three examples that represent distinct faces of symbiosis. The first one is endosymbiosis, which was crucial in the origin of eukaryotic cells. In fact, we can consider eukaryotes as symbiotic mergers forged via cooperative interactions by progressive physical integration and endosymbiotic gene transfer [58]. The second example is mutualism. The pea aphid, *Acyrthosiphon pisum*, has several species of bacteria (*Buchnera, Rickettsiella* and *Hamiltonella*) that live in its cells [59]. These insects rely on *Buchnera* to provide nutrients, on *Rickettsiella* for color changing of the aphid, and on *Hamiltonella* to defend the insect against wasp infection with the help of a lysogenic phage [60]. So there is a multiple cooperation to feed and protect the aphid from wasps and to provide a home for the bacteria that keep the phage rented. The third example is the holobiont. Holobionts are multicellular organisms that have co-evolved with complex consortia of viruses, bacteria, fungi and parasites, known collectively as the microbiota [61]. Changes in the composition of microbiota can influence metabolism, digestion, immunity, neuronal activity and behavior, and also they are associated with multiple diseases [62]. This tight host-microbiota cooperative relationship challenges the concept of individuality by a conception congruent with symbiotic associations as the evolutionary unit [63].

Multicellularity is one of the major evolutionary transitions in the history of life [64]. The key feature of multicellularity is the cooperative thrust because it provoked not only an increase in the diversity of species that colonized distinct biotopes but also in the complexity or organisms (organization of the different cell types in tissues and organs during morphogenesis). This commandment would be the main force to give rise to animals, plants, fungi, and algae. So that for this increase in the complexity to be functional, multicellular organisms needed new integration or coordination mechanisms to ensure cooperation amongst all the cells, tissues and organs. The oldest way of communication between cells was chemical (chemotaxis, quorum sensing) and physical through cell junctions. Later on they appeared more complex systems of cellular communication such as signal transduction (that implies a receptor that receives the signal, a transduction mechanism involving second messengers, and finally a cellular or physiological response), electric signals, proteins of the extracellular matrix, and the existence of specialized tissues for coordinating the organism such as the endocrine and nervous systems [65].

Interestingly, it has been suggested that the emergence of multicellular organisms was not a “difficult problem” in evolution and that multicellular complexity may evolve more readily that previously thought [66]. Once the first multicellular organisms began to exist, two new cellular processes had to emerge quickly, otherwise the group of cells would be just a colony: the genetic control of development (growth and morphogenesis) to ensure the continuity of multicellular life, and the process of cell differentiation that gave rise to tissues and organs. Furthermore, the increase in the complexity of multicellular organisms provoked the appearance of coordination mechanisms to ensure cooperation amongst all the cells, tissues and organs. The cooperative thrust, the inexorability principle, the evolutionary imperative and the conservative rule were acting together in the generation of multicellular life.

We can conclude that living entities have the necessity to cooperate to survive and evolve (for this reason I talk about the cooperating thrust) and that each ecosystem is a giant cooperative network, a place filled up with cooperative interactions. 

## Concluding remarks: the endless race

Life must struggle against the second law of thermodynamics, against the tyranny of time. The living beings always loose this battle against entropy and time: there is no immortality and death is the end of the tyranny of time, the victory of entropy. However, there is a paradox: if all organisms had to die, life would disappear, and we know that this does not happen; on the contrary, since life emerged on Earth, millions of years ago, it began an endless succession of new living forms that we recognize as the evolution. Reproduction and evolution are responsible for the victory of life over entropy and time.

I believe that the seven commandments are fundamental to understand the vital phenomenon. Using them we can find a fairly satisfactory answer about the origin of life, the generation of biodiversity and the evolution of ecosystems (manuscripts in preparation). In the origin of life, the beginning of history, the first three commandments could have been fundamental, as well as the laws of physics and chemistry of course. I believe that before the first cell originated there had to be an ordered structure, at least formed by a membrane and a set of molecules inside. From this ordered prebiotic structure the first cell eventually emerged when it acquired the ability to divide. In this sense, the origin of life is related to the vital order. It is also related to the second commandment because at the molecular level there is a chemical determinism that causes the essential metabolic pathways to occur, even in the absence of enzymes, or proteins to adopt their ideal tertiary structure. Without this “inexorability” the first cell could not originate. We know that transcription and translation, the reading of the genetic message can occur even in the absence of a cellular structure; this form is related to the third commandment that tells us about the flow of genetic information.

**Figure 4.**
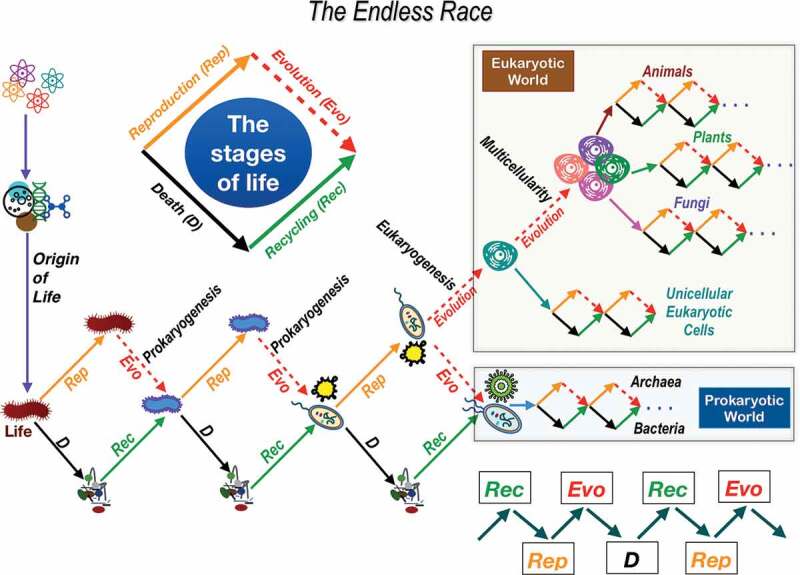
The endless race. After life originated on earth, it began the evolutionary process by becoming an endless race without a finish line and the rules that governed this race were the commandments. Every organism has to go through the four stages of life: reproduction (Rep), evolution (Evo), death (d) and recycling (Rec) of the disorganized living matter. During the entire evolutionary process, first prokaryogenesis (the generation of the initial bacteria and archaea) took place, followed by eukaryogenesis as a consequence of endosymbiotic events. Some single-cell eukaryotic cells cooperated to give rise to multicellularity and from there give rise to animals, plants and fungi. The eukaryotic and prokaryotic worlds have interacted with each other and with the acellular world (virus) and its environment ever since.

The thread of this history is the successive stages of life that have always included four closely related processes: reproduction, evolution, death and recycling (Figure 4). When living beings reproduce, a new order is created and the timer of life is set to 0. The Vital Order is the key commandment in this fundamental biological process. But organisms must change to adapt to the new scenarios (competition, environmental changes, energy sources, new species) otherwise they will disappear. This is what we call evolution and is responsible for the creation of new species. During the evolutionary process, there were so special and extraordinary events: the prokaryogenesis or generation of bacteria and archaea that make up the prokaryotic world, the eukaryogenesis or genesis of eukaryotic cells, and the multicellularity that gave rise to plants, fungi and animals; unicellular and multicellular eukaryotic organisms make up the eukaryotic world. In the evolutionary process the second, fifth, sixth and seventh commandments are playing an essential role. Death is necessary for the survival of future generations. But death also gives way to life through the recycling of organic and inorganic matter that is generated when a living being dies. Recycling feeds the creation of new life. The four stages of life are involved in an endless race of biological events toward an unsettled arrival. Life is like an endless relay race in which living beings are the runners, who change as the race goes on, and the commandments are the rules.

## Declarations

No funding supported this work. There are no conflicts or competing interests.
